# Complete genome sequences of *Lactococcus lactis* D1_2, a bacterium with antimicrobial properties isolated from peat soil

**DOI:** 10.1128/MRA.00680-23

**Published:** 2023-11-07

**Authors:** Priscilla Isaac, Prasanna Mutusamy, Lee Su Yin, Yap Jing Wei, Faezah Mohd Salleh, Muhammad Abdul Latiff bin Abu Bakar, Sivachandran Parimannan, Heera Rajandas

**Affiliations:** 1 Centre of Excellence for Omics-Driven Computational Biodiscovery (COMBio), AIMST University, Bedong, Kedah, Malaysia; 2 Centre of Research for Sustainable Uses of Natural Resources (SUNR), Faculty of Applied Sciences and Technology (FAST), Universiti Tun Hussein Onn Malaysia, Pagoh Higher Education Hub, Muar, Johor, Malaysia; 3 Department of Biosciences, Faculty of Science, Universiti Teknologi Malaysia, Johor Bahru, Johor, Malaysia; 4 Environmental Management and Conservation Research Unit (eNCORe), Faculty of Applied Sciences and Technology (FAST), Universiti Tun Hussein Onn Malaysia, Pagoh Higher Education Hub, Muar, Johor, Malaysia; 5 Center for Evolutionary Hologenomics, Globe Institute, University of Copenhagen, Copenhagen, Denmark; University of Maryland School of Medicine, Baltimore, Maryland, USA

**Keywords:** WGS, *Lactococcus lactis*, peat swamp forest

## Abstract

*Lactococcus lactis* is a beneficial lactic acid bacterium commonly studied for its probiotic properties and role in dairy production. Here, we present a complete genome of *Lactococcus lactis* D1_2, isolated from peat swamp forests. To discover the potential antimicrobial properties, the complete genome of the strain was sequenced and analyzed.

## ANNOUNCEMENT

*Lactococcus lactis*, generally known as lactic acid bacteria (LAB), is a Gram-positive bacterium that is used as a starter culture in the production of dairy products such as yogurt, milk, and cheese. LAB has been employed as probiotics due to their antimicrobial properties, such as the production of organic acids (mainly lactic and acetic acids) and ribosomally synthesized antimicrobial peptides that are referred to as bacteriocins (1). LAB with a broad spectrum of antibacterial properties is extensively used for prolonging the shelf life of food and preventing spoilage and foodborne pathogenic bacteria (2).

Here, we report the complete genome of the *Lactococcus lactis* D1_2 strain, which was isolated in 2022 from peat soil collected from Hutan Simpan Ayer Hitam Utara, Johor, Malaysia (N 02°04.4470′, E 102°49.0151′). One gram of soil was resuspended in 10 mL of tryptic soy broth (TSB); it was serially diluted and cultured on tryptic soy agar to obtain the strain. The isolate was aerobically grown in TSB medium overnight at 30°C, and the genomic DNA was extracted using the NucleoSpin Tissue Kit (MACHEREY-NAGEL) following the manufacturer’s instructions.

Unsheared and nonsize-selected DNA was used for the preparation of libraries (SQK-NBD112.24 native barcoding kit, Oxford Nanopore Technologies) that were sequenced on the SpotON flow cell (R10.4.1) using the MinION device, and the basecaller used was Guppy v6.5.7 (high-accuracy model). The raw sequencing data of 17,598 reads with a read length N_50_ of 5,627 were trimmed using Porechop v0.2.4 (3), and the quality of the reads was evaluated by Nanoplot v1.41.6 (4). After trimming, the remaining 17,545 reads were assembled *de novo* using Flye v2.9.1-b1780 (5). This resulted in a single circular contig, as indicated by Flye, without an overlap at the contig ends (verified by local alignment). The genome was not rotated and subsequently polished using Medaka v1.8.1 (https://github.com/nanoporetech/medaka). Genome annotation was carried out using the NCBI Prokaryotic Genome Annotation Pipeline v6.5 (6). Default parameters were used for all the software tools involved in the analysis, unless otherwise specified.

The final genome assembly yielded a circular chromosome of 2,457,726 bp, an N_50_ length of 2,457,726, a GC content of 35.13%, and 23× genome coverage. The genome contains a total of 2,647 genes made up of 2,142 intact coding sequences, 19 rRNAs, 60 tRNAs, 4 noncoding RNAs, and 422 pseudogenes. *Lactococcus lactis* subsp*. lactis* Il1403 was found to be its closest neighbor using Similar Genome Finder Server (7). As measured by fastANI v1.1.0 (https://proksee.ca/tools/fastani), the average nucleotide identity between these two strains is 98.13%.

Antibiotic biosynthetic gene clusters of strain D1_2 were predicted using antiSMASH v7.0.0 (8) by using default options, “relaxed” detection, and “all on” extra features. Analysis with the antiSMASH tool uncovered secondary metabolite BCGs in five regions, including one bacteriocin, one betalactone, one T3PKS, and two RaS-RiPP. Similarly, bacteriocin was detected through BAGEL4 (9) analysis software. Our findings suggest that the D1_2 strain has the antimicrobial activities of bacteriocins that can be employed as a food preservative and as an alternative to traditional antibiotics. This suggests that peat swamp forests can be explored as potential sources of beneficial bacteria.

## Data Availability

The complete genome sequence of *Lactococcus lactis* was deposited at NCBI GenBank under the BioProject accession number PRJNA949442, the Biosample accession number SAMN34062271, and the GenBank accession number CP121454.
